# Biochemical mediators during active and retentive phases of tooth movement in orthodontic patients—a scoping review

**DOI:** 10.3389/froh.2025.1681304

**Published:** 2025-11-17

**Authors:** Venkateswaran Ananthanarayanan, Sridevi Padmanabhan

**Affiliations:** Department of Orthodontics and Dentofacial Orthopedics, Sri Ramachandra Dental College, Chennai, India

**Keywords:** mediators, relapse, tooth movement, bone remodelling, cytokines

## Abstract

**Introduction:**

Crowding or spacing of teeth will impair oral hygiene maintenance and therefore lead to poor oral health and nutrition. Orthodontic treatment aligns the teeth and establish a proper occlusion, both of which are essential in the integrity of oral health and nutrition. Despite advancements in orthodontic treatment, relapse (teeth returning to the previous position) remains a major challenge. Previously, collagen turnover was considered the main factor, but aligns studies suggested extracellular matrix proteins such as tyrosine protein kinase (TEC protein) play a more significant role due to their exclusive presence during the retention phase. While extensive research exists on OTM, few studies have explored biochemical mediators during retention. The primary objective of this scoping review is to identify biochemical mediators at different timelines during OTM and relapse to consolidate findings and address gaps so that orthodontists may attempt to alter the mediators thereby restoring oral health and nutrition.

**Method:**

This scoping review complied with the PRISMA-ScR guidelines. The search terms used were MESH terms and Boolean terminology. The search was conducted until July 2023 across five databases; PubMed, Scopus, Medline, Embase, and Google Scholar, including gray literature and unpublished data. The resulting numbers of articles (120) were chosen for the scoping review after matching with the framed inclusion and exclusion criteria (distributed as 113 and 7 studies for active and retentive phases respectively). Each reviewer stored the retrieved list of articles in separate folders designated for each database. The two reviewers resolved discrepancies through discussion. Any points of disagreement or conflict in the conducted search were escalated to the third senior reviewer, whose judgment was final.

**Results:**

The extraction of relevant data was independently performed by the two reviewers. The following data were analyzed: author name, journal, year of publication, type of study, sample size, sample site, type of biomarker assessed, and stage of orthodontic treatment. Queries pertaining to a particular study were clarified by contacting the lead author. The data were compiled individually by each reviewer into a draft chart and then discussed to reach a consensus. These data were then shared with a third senior reviewer to streamline and finalize the data.

**Conclusions:**

The literature on biomarkers of tooth movement is exhaustive. However, studies on biomarkers during the retention phase are limited, and more exploration is needed.

**Systematic Review Registration:**

https://osf.io/sh6u5.

## Introduction

### Rationale

In recent decades, the mechanism of orthodontic tooth movement has been limited to the pressure tension theory, wherein, upon orthodontic force, there is bone resorption on the pressure side and bone apposition on the tension side, thus initiating the remodeling process to move teeth (1). During these events, biological mediators are released by the cells involved in remodeling. The predominant mediators are cytokines, growth factor enzymes and hormones, which initiate a cascade of orchestrated events leading to alterations in the nuclear protein matrix and eventually gene modulation, resulting in mechanotransduction. The most commonly expressed and documented cytokines include interleukins (ILs) (IL-1α, IL-1β, IL-1RA, IL-8, IL-2, IL-6, and IL-15), tumor necrosis factors (TNFs), interferons (IFNs), growth factors (GFs), and colony stimulating factors (CSFs). IL-1β, IL-2, IL-6, IL-8 and TNF-α are some of the cytokines reported by authors to be elevated during the first month of orthodontic force application (2–8). The earliest identified marker of bone resorption is IL-1β, followed by prostaglandin E2 (PGE2) (9, 10), nitric oxide, IL-6, and other inflammatory cytokines. An increase in the concentration of matrix metalloproteinases (MMPs), such as MMPs 1, 2, 3, 8 and 9, was also reported by other authors (11–15). Ariffin SHZ et al. reported elevated tartrate-resistant acid phosphatase (TRAP) levels during the initial days of active orthodontic tooth movement (16). Elevated myeloperoxidase (MPO) is observed at the second hour of activation of a fixed orthodontic appliance (17).

Despite advances in orthodontic appliances and treatment modalities, relapse remains an issue. Earlier, it was believed that the major contributor to relapse was collagen turnover in the gingiva and periodontal ligament. This notion was subsequently disproved by Maltha et al. (18) and Nakanishi (19), who reported that the rate of collagen fiber turnover in gingival and periodontal tissues were high at 7 days, which was too short to contribute to long-term relapse. Hence, other extracellular matrix proteins have been suggested to be responsible. Although many studies have explored the biological mechanisms and markers of orthodontic tooth movement, very few studies have investigated the biochemical mediators involved during the retentive phase of orthodontic treatment. Several animal studies have evaluated RANKL, Alkaline Phosphatase (ALP) and TRAP levels (20, 21). Few human studies have evaluated biochemical markers during relapse at a single time point. In 2019, Awang-Kechik et al. (22) determined the levels of biochemical markers during retention after treatment with a fixed appliance. Among the many markers studied, tyrosine–protein kinase (TEC protein) was detected only during the six-month retentive phase but, surprisingly, not during active tooth movement.

The literature on the mediators of OTM and relapse is numerous and scattered, i.e., a combination of animal and human studies and *in vitro* or *in vivo* studies with many other influential factors that play a role in the expression of these mediators. In addition, studies on the molecular events and biochemical mediators that prevail during relapse are limited and in the primitive stage. Despite the abundant literature on the molecular events leading to orthodontic tooth movement and the relatively few reports on relapse, the extent of the roles of the mediators across the various phases of orthodontic treatment, including retention, is still unclear. For these reasons, a scoping review was deemed mandatory to streamline the events and to identify the lacunae in the literature.

### Objective

To identify biochemical mediators at different times during orthodontic tooth movement and during relapse. The broader research question of this review was “What are the biochemical mediators of tooth movement in orthodontic patients during and after orthodontic treatment?” The sub question was “Are the biochemical mediators that are expressed during orthodontic tooth movement similar to those expressed during relapse?”

## Methods

### Protocol and registration

The protocol was drafted on the basis of the PRISMA-ScR guidelines constructed by Tricco et al. (23) and was registered in the Open Science Framework (https://osf.io/sh6u5).

### Eligibility criteria

The inclusion criteria were designed on the basis of the population concept context (PCC) format recommended by Tricco et al. (23) and are outlined in Table 1 below.

**Table 1:** Inclusion and exclusion criteria based on the PCC format.

Domain	Inclusion criteria	Exclusion criteria
Population	Human or animal studies where orthodontic tooth movement is initiated where control is the baseline levels or antagonistic or contralateral tooth *in vitro* studies	Orthodontic patients in which markers of tooth movement was not evaluated
Concept	Studies in which the following are assessed; mediators of tooth movement like inflammatory markers, cytokines, enzymes, proteins, RANK RANKL	Studies which have not focused on qualitative or quantitative assessment of biochemical mediators of orthodontic tooth movement
Context	Studies in which markers are evaluated during and/or after orthodontic tooth movement	Studies in which tooth movement is not executed

### Information sources

The search was conducted until July 2023 across five databases, namely, PubMed, Scopus, Medline, Embase, and Google Scholar. Studies published only in English were considered. The searches were rerun prior to the final analysis. The gray literature and unpublished data, if retrieved, were also included.

### Search strategy

**Figure 1:**
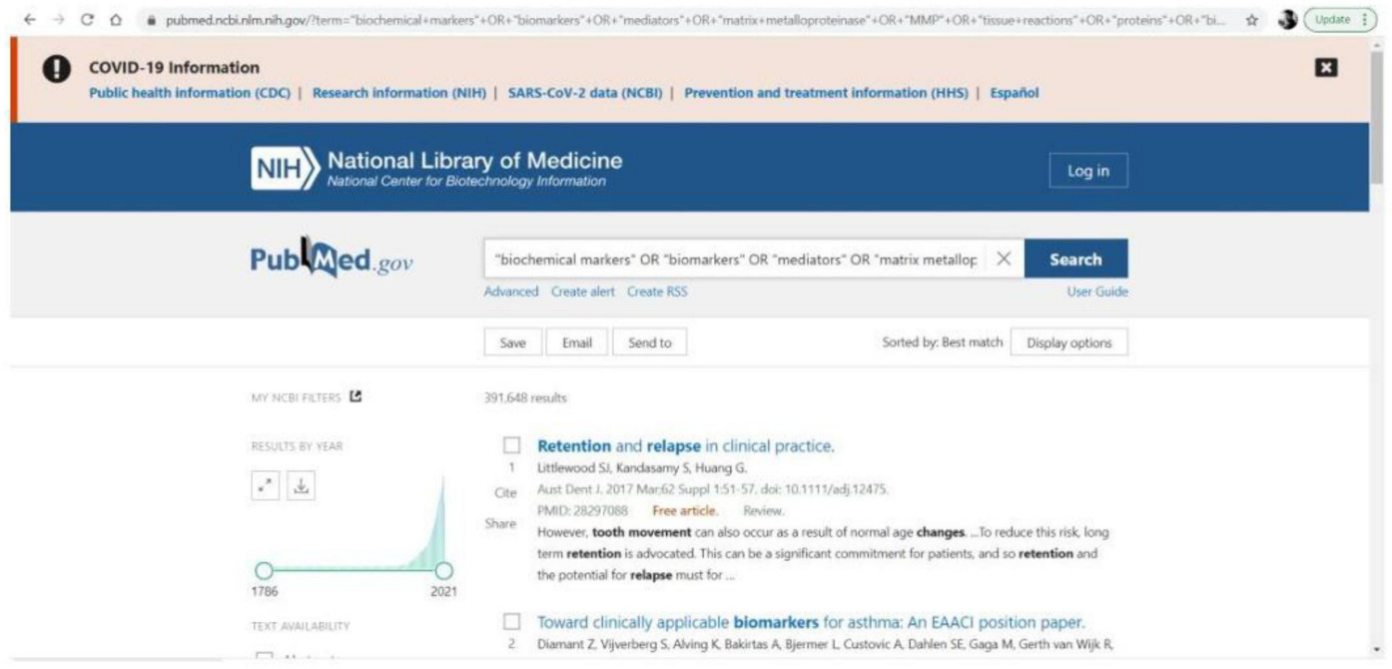
Screenshot of the search strategy employed in PubMed.

The search terms used were the following MeSH terms and Boolean terminology: “biochemical markers” OR “biomarkers” OR “mediators” OR “tissue reactions” OR “proteins” OR “biology” OR “molecular changes” OR “cytokines” OR “enzymes” OR “GCF” OR “saliva” AND “force” OR “tooth movement” OR “teeth movement” OR “relapse” OR “retention”. The search words were reviewed with the Peer Review of Electronic Search Strategies (PRESS) checklist [McGowan et al. (24)] Search words were initially selected for PubMed and were later modified to suit the preferences of the other databases (Figure 1).

### Risk of bias & quality considerations

Consistent with the guidance of scoping reviews, we did not deem it mandatory to perform a risk of bias assessment prioritising a comprehensive identification and pooling of evidence over formal risk of bias appraisal. However, the quality of grey literature was assessed using the AACODS checklist (Authority, Accuracy, Coverage, Objectivity, Date, Significance). Both the reviewers appraised the included data but did not exclude them. The search was conducted across five indexed data bases to mitigate publication bias and strengthen the quality of included articles. Gray literature such as theses, conference proceedings, preprints, and trial registries was also backed up with backward/forward citation searches and deduplicated records in order to reduce positive—result bias.

### Selection of sources of evidence

The publications obtained from the electronic search were imported into Mendeley (https://www.mendeley.com), with which duplicates were removed. The first level of screening excluded articles with irrelevant titles. The abstracts of the remaining articles were read and excluded if not relevant. The resulting number of articles was chosen for the scoping review after matching with the framed inclusion and exclusion criteria. Venkateswaran Ananthanarayanan (VA) stored the retrieved list of articles at each screening level in separate folders designated for each database in the Mendeley software. VA resolved discrepancies through discussion with the senior reviewer Sridevi Padmanabhan (SP) whose judgment was considered final.

### Data charting process

VA developed a table to extract the relevant information from the included sources of evidence. The data were filled in by VA and crosschecked by the SP. Both reviewers discussed the results, and the form was constantly updated.

### Data items

The following data were analyzed and charted from the selected articles: author name, journal, year of publication, type of study, sample size, sample site, characteristics of intervention (stage of orthodontic treatment), and outcome measures (type of biomarker assessed). In the process, any queries pertaining to a particular study will be clarified by contacting the lead author.

### Synthesis of results

The data were compiled individually by VA into a draft form and then discussed with SP to reach a consensus. These data were then shared with SP to streamline and finalize the data. A detailed explanation of the studies across the above domains of assessment was provided. The results are presented via descriptive statistics such as percentages, tables, charts and flow diagrams, as appropriate. Narrative analysis was used to summarize the findings of the review.

## Results

### Selection of sources of evidence

**Figure 2:**
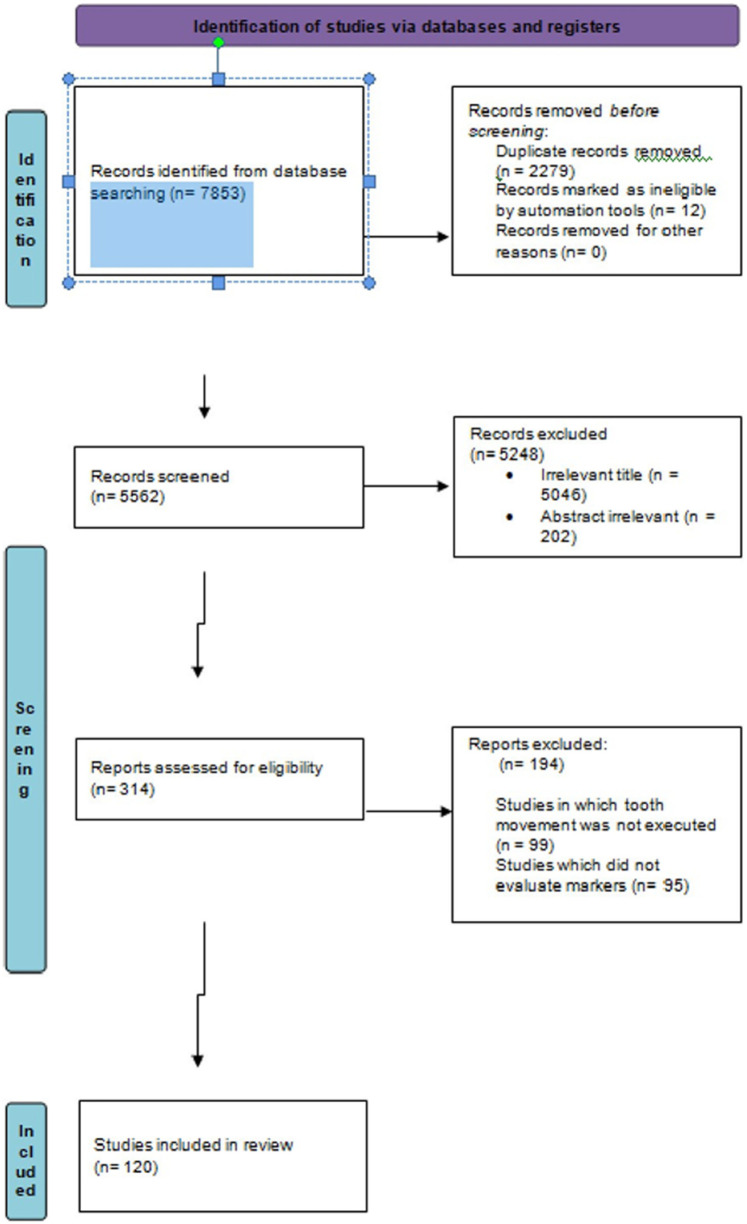
PRISMA flow chart depicting the search strategy.

The search was conducted in accordance with the PRISMA 2020 flow diagram and is illustrated in Figure 2 (PRISMA flowchart depicting search strategy) below. Of the 120 included studies, 113 evaluated biomarkers during active tooth movement, whereas 7 studied molecular changes after active tooth movement, i.e., the retentive phase.

### Characteristics of the sources of evidence

The existing sources of evidence can be categorized into those related to active tooth movement and those related to the retention phase. The categories in which each study was described were the author and year of study, sample characteristics, characteristics of the intervention, and follow-up duration. The domains of description with respect to the studies of active tooth movement have been described by Kapoor et al. (25, 26), Vansant et al. (27), Alhadlaq (28), Allen et al. (29) over the past decade. Similar domains were used to describe the characteristics of the studies of biomarkers during relapse and are depicted in Table 2.

**Table 2:** Characteristics of the studies pertaining to biomarkers during retention/relapse.

Author, year	Study design	Sample characteristics	Characteristics of intervention (Stage and duration of Orthodontic treatment)	Follow up duration
Reitan (30)	Review	Orthodontic patients; age not specified	Not mentioned	Not specified
Murell et al. (31)	Prospective, animal split mouth study	28 male Sprague‒Dawley rats	Orthodontic appliance to mesially tip maxillary 1st molar for 2 weeks	21 days
King et al. (21)	Prospective animal study	44 male Sprague‒Dawley rats	40 g of orthodontic force for 16 days	14 days
Burke et al. (32)	Cross sectional study	19 orthodontic patients	Separators for 24 h	24 h(Immediately after removal of separators)
Franzen et al. (20)	Prospective animal study	Forty male Wistar rats	Orthodontic force with a closed coil spring for 10 days	21 days
Perinetti et al. (33)	Split-mouth longitudinal study	23 subjects (15 girls, 8 boys; mean age, 9 years)	Rapid maxillary expansion	6 months post expansion
Awag-Kechik et al. (22)	Prospective cohort clinical study	25 patients (15 experimental, 10 control). 18 female and 7 males.	Orthodontic treatment duration not mentioned	6 months post orthodontic treatment

### Synthesis of results of individual sources of evidence

For ease of comprehension, the results of this scoping review were broadly tabulated into studies that focused on the changes occurring during orthodontic tooth movement and those that evaluated the changes during the retentive phase of orthodontic treatment (Table 3).

**Table 3:** Classification of studies that evaluated changes during and after orthodontic tooth movement.

Outcome measure	Phase of orthodontic treatment	
	Active	Retentive
Tissue changes	Gingival fibers (34–41)	Reitan & Murell in 1967 & 1996 (30, 31)
	Periodontal fibers (19, 42–51)	
	Alveolar bone (42–51)	
Biomarkers	Enzymes (MMPs, ALP, TRAP, LDH, AST, Cathepsin, beta glucuronidase) (11–17, 52–67, 75–77)	Markers of alveolar bone remodeling;
	Salivary proteins (29)	Animal studies (20, 21)
	Cytokines (2–8)	Human studies (33)
	Prostaglandins (9, 10)	Salivary proteins; Human studies (22, 32)
	ECM & Cell membrane receptors (68, 69); Neuropeptides (70, 71)	

### Biochemical markers of orthodontic tooth movement

The literature on the molecular basis of orthodontic tooth movement is exhaustive, and four systematic reviews have been conducted by Kapoor et al., Vansant et al., Alhadlaq and Allen et al. (25–29).

Kapoor et al. conducted systematic reviews exclusively on cytokine and enzyme expression during orthodontic tooth movement in 2014 and 2019, respectively. Most of the cytokines are released during the first 24 h and gradually decrease after the application of orthodontic force, suggesting that remodeling occurs only during initial tooth movement. These levels increase immediately after every reactivation. The RANKL/OPG ratio increased during initial tooth movement, especially in growing subjects, indicating the potential for accelerated tooth movement immediately after an orthodontic force, which is amplified in young patients. However, none of the studies showed a clear distinction in biomarker expression between the sexes, and several confounding factors were either ignored or not explored in the literature (25). The enzymatic expression varied depending upon the stage and site of orthodontic force application. The compression sites presented early increases in the levels of MMP1, MMP2, TIMP1, and MMP9 from 1 to 4 h and late peaks in the levels of TIMP2, TRAP, and AST after 7 days, 4–5 weeks and 8–12 weeks, respectively. Tension sites showed a significant increase in ALP after 7 days and in MMP1 between the first 1st and third hours (25, 26).

Vansant et al. published a systematic review on the biological mediators of orthodontic tooth movement in 2018. In addition to the human and animal studies, they also reported few *in vitro* studies and realized that the setup of the latter was considerably different, which raised concerns about the validity of the studies (27). Osteoclast formation and recruitment are induced by factors such as IL-6, TNFα and chemokines (especially CCL2, CCL3 CCL5 and CXCL2), which are increased within the first week of OTM. Bone apposition at tension sites is mediated by runt-related transcription factor (RUNX2), osterix (OSX) and osteocalcin (OCN), which are expressed *in vivo* within the first 2 weeks (27). The MMPs and cathepsins increased on the compression side within 1 week of OTM and decreased by the 2nd week. These enzymes help degrade the extracellular matrix, which is then replaced by a new matrix. Transforming growth factor (TGF)-β levels increase gradually until 4 weeks, which is consistent with the synthesis of new extracellular matrix (27).

Allen et al. performed a systematic review of salivary protein biomarkers during orthodontic tooth movement and reported that these biomarkers are useful not only for studies pertaining to tooth movement but also for future research on their role in root resorption (29).

#### Tissue changes and biochemical markers during orthodontic retention

The results of the included studies are described in Table 4.

**Table 4:** Description of the results of the studies included in the systematic review demonstrating tissue and biochemical changes during orthodontic retention.

Author, year	Type of study	Outcome measure	Site of expression &/or method of assessment	Results
Reitan (30)	Review	Principle fibers of the periodontium especially supraalveolar fibers	In different areas of the root based on the type of tooth movement	After derotations, principle fibers returned to original positions in a few days; supraalveolar fibers took approximately 232 days
Murell et al. (31)	Animal	Number of blood vessels	Periodontal ligament	12–50 micron diameter: Numbers around middle third of root increased and peaked in 3 days and declined over the next 2 weeks 51–200 micron diameter: Numbers around the cervical third of root decreased in the first two days and increased the next two days after which returned to baseline levels
King et al. (21)	Animal	Histological: osteoclast numbers per millimeter of bone surface, osteoclast surface percentage, osteoblast surface percentage, mineralizing surface percentage, mineral apposition rate, bone formation rate, and percentage of resorbed root surface	First molar sections for histomorphometry and sera and aparadental alveolar bone for the biochemical assay	Histological: Mesial end of molar—osteoclast number and surface % elevated 3.5 to 4 times on day 1 and returned to baseline by day 5. Distal end of molar—No time- related treatment effects on osteoclast number and surface %
		Biochemical: Bone phosphatases (acid phosphatase, TRAP, alkaline phosphatase), bone remodeling parameters (osteoblast and osteoclast percentage)		Biochemical: Significant elevations in acid phosphatase and TRAP in the treated tissues at days 5, 7, and 10. No time- or treatment-related differences in serum alkaline phosphatase activities
Franzen et al. (20)	Animal	Bone architecture: Bone mineral density, percent bone volume, bone surface density, total porosity.	Micro ct of alveolar bone RTPCR to evaluate bone formation markers runx2, *COL1A1*, ALP, OCN and bone resorption markers H + -ATPase, TRAP and RANKL	Relapse upto 93% by 21 days
				1st day—73% relapse (rate of 140 microns/day
				2nd to 21st day—93% relapse (Rate almost negligible)
				Microct evaluation: Tissue mineral density decreased During OTM and returned to baseline levels 3 days after removal Biomarkers—ALP, OCN, COL1A1, Runx2—No significant correlation with relapse
				RANKL—Decrease after appliance removal till baseline by 21 days
				TRAP—Insignificantly increased till 7 days after appliance removal then returned to baseline levels by 21 days
Perinetti et al. (33)	Human	Periodontal health: Supragingival plaque, bleeding on probing, pocket depth	Gingival Crevicular fluid	Periodontal Health: Plaque & bleding on probing: No significant differences between test teeth (TT) (maxillary first molar) and mandibular first molar (CT) (control teeth) at T1 (3months) and T2 (6 months) post expansion
		Alkaline phosphatase		Pocket depth: Significant differences between TT & CT at T1 and T2
				Alkaline phosphatase: Significant increase at the TT site Significant increase at T1 & T2 at the TT site
Burke (32)	Human	Total secretory proteins and cyclic adenosine monophosphate (c- AMP)—dependant protein kinase subunit (RII)	Saliva and Gingival Crevicular fluid; Electrophoresis and Western blotting	Electrophoresis: Total protein concentration—No significant increase in GCF and saliva (Increased in some and decreased in other patients)
				Western blotting: c-AMP dependant protein kinase subunit (RII)—No significant increase in GCF and saliva (Increased in some and decreased in other patients) 50-to-55 kd bands, characteristic of RII, disappeared, while a fragment in the 20 to 30-kd range increased considerably after separator placement
Awag- Kechik et al, (22)	Human	Protein profile	Saliva; Liquid Chromatography‒Mass Spectrometry (LC‒ MS)	Little's Irregularity index: Significant difference in irregularity in mandibular arch 6 months into retention phase Protein profile: Active phase—146 proteins Retentive phase—135 proteins 15 proteins expressed in both active and retentive phase 1 protein (tyrosine protein kinase) expressed only in retentive phase

#### Tissue changes during retention phase

In 1967, Reitan (30) was one of the first people to observe changes in the PDL of teeth that experienced orthodontic force. Clinical and histologic observations were made both during and immediately after orthodontic treatment. In terms of relapse, he attributed it to the muscular imbalance caused by moving the dentition. In addition, another major contributing factor was the rearrangement of the principal fibers of the gingiva and PDL. He explained that the rearrangement of the principle fibers might not take long, whereas the supra-alveolar fibers might take a longer time and remain stretched. He concluded that this might have a greater role in the relapse of orthodontic treatment. Murrell et al. (31) reported that during two weeks of orthodontic force application, the number and density of blood vessels increased. Immediately after the removal of orthodontic force, there was an immediate decrease in the blood vessel profile, after which it increased and then returned to the baseline levels by the end of 3 weeks posttreatment.

#### Biochemical markers during retention phase markers of alveolar bone remodelling

##### Animal studies

King et al. (21) studied alveolar bone turnover after orthodontic tooth movement in rats. Turnover was evaluated with both histomorphometric and biochemical changes in the oral tissues. One group was subjected to 40 g of force applied to move the maxillary first molars mesially, whereas the other group was subjected to all procedures except spring placement to actively move the tooth. The treatment was active until 16 days, after which it was discontinued. The following parameters were assessed at both the mesial and distal roots of the maxillary first molars in both groups: bone phosphatases (acid phosphatase, TRAP, alkaline phosphatase), bone remodeling parameters (osteoblast and osteoclast percentage) and root resorption (percentage of resorbed root surface). The biochemical changes revealed a sustained increase in the level of bone phosphatases at the mesial roots (previously pressure sites). In 2014, Franzen et al. were the first and probably the only authors who have studied relapse three-dimensionally via micro-CT. Orthodontic force was applied to rat first molars to move them mesially, and the biochemical markers of alveolar bone remodeling, both during and after orthodontic treatment, were estimated. One day after appliance removal, the molars started to relapse; the levels of ALP, osteocalcin, and COL1A1 started to increase. By the end of 3 weeks, the levels had returned to baseline. There was no significant correlation between TRAP, RANKL or HTPase and relapse.

##### Human studies

Perinetti et al. (33) estimated the levels of ALP in the GCF of teeth, which are used to support the appliance for rapid maxillary expansion required in prepubertal patients with constricted maxillary arches. The samples were procured from the tension sites of the teeth at 3-month and 6-month intervals. The results revealed that there was an increase in ALP activity at the tension sites of the tooth PDL, which decreased by the end of the 6 months postexpansion. This was a split-mouth longitudinal study in which the antagonist teeth served as the control. The control side also presented increased ALP activity, but it was not greater than that of the experimental side. The authors concluded that bone formation postexpansion might be complete by the end of 6 months postexpansion, thereby indicating the need for retention for a minimum of 6 months postexpansion.

#### Salivary proteins

##### Human studies

Burke et al. (32) evaluated the expression of secretory proteins in the GCF and saliva from patients for whom separators had been placed. The separator placement was considered to be the source of force application. The samples were collected before and 1 day after separator placement, and total secretory proteins and cyclic adenosine monophosphate (c-AMP)-dependent protein kinase subunit (RII) were measured via electrophoresis and western blotting. The results revealed that there was no significant increase in the total protein concentrations in the GCF or saliva. However, mechanical strain causes a significant increase in the RII subunit, indicating that a systemic response through the AMP signaling pathway might be initiated. Awang-Kechik et al. (22) conducted one of the few protein profile analysis studies in patients during retention after orthodontic treatment. The retention phase was 6 months, and the protein profile was analyzed via liquid chromatography‒mass spectrometry (LC‒MS). Relapse was quantified via Little's Irregularity Index. The results revealed 146 proteins that were expressed in the control group (untreated) and 135 proteins expressed in the retention group. The protein that was detected only during the retention phase and not in the control was the immune response protein tyrosine-protein kinase (TEC). This result encouraged the authors to conclude that TEC protein might be a good potential biomarker for predicting relapse.

Although a certain degree of heterogeneity across the included studies remains, a relatively consistent pattern of biomarker expression was observed across the included studies. During **active tooth movement,** there was a transient increase in the ALP at tension sites within the 1st 1–2 weeks followed by the increase in OCN (2–4 weeks later).

**During the retention phase,** animal model studies reveal an initial increase in the ALP/OCN/ COL1A1 immediately after debonding. This is followed up with a return to the baseline levels by 3 weeks.

## Discussion

This systematic review aimed primarily to thoroughly review the literature, which has explored the changes that occur during the active and retentive phases of orthodontic tooth movement. The biomarkers that have been studied include cytokines; enzymes; RANK, RANKL, OPG, MMP, and TRAP; salivary proteins; osteocalcin; TGF-β; ALP; TNFa; cathepsin B; epidermal growth factor; beta-2-microglobulin; glycosaminoglycans; PGE2; LDH; AST; neurotransmitters; metabolites of arachidonic acid; and growth factors, among many others. Among the mediators, IL-1β and TNF-α have been the most studied (78). During active tooth movement, studies have shown a common consensus on the increased levels of bone resorptive mediators, which peak by the 24th hour and return to baseline levels within 3 weeks of orthodontic force application. However, the pattern is not sustained across the studies and is rather reversed in some studies. This could be explained by the fact that the sites of compression and tension are in close proximity.

During the first days of force application, the levels of apoptotic mediators such as heat shock protein, caspases, BCL-2-associated X protein (BAX), B-cell lymphoma 2 (BCL-2), death domain-containing protein (CRADD) and the Bcl-2-associated death promoter (BAD) tend to increase in osteoblasts after compressive stimulation (27). There was also a significant increase in the GCF levels of the MMP inhibitors TIMP-1 at 4 h and TIMP-2 after 7 day during canine retraction (26). Both of these findings indicate a lag phase of tooth movement. Apoptotic signals are then followed by cell strain signals such as connexin 43, marking mechanotransduction, which eventually activates intracellular signaling pathways, leading to cell differentiation and the activation of osteoclastic precursors. This initiates the RANKL—OPG pathway, wherein there is an increase in the RANKL protein levels at compression sites and an increase in the OPG levels on the tension side immediately after force application (27, 72). This finding was not consistent across the studies; rather, the sRANKL/OPG ratio seemed more closely associated with OTM (29). An interesting observation was made in a comprehensive salivary protein analysis by Awang-Kechik et al., who compared protein expression in the active and retentive phases of orthodontic treatment. There was the presence of an immune system protein [identified as tyrosine-protein kinase TEC (TEC protein)] in the retention phase that was not expressed in the active phase. This protein, in addition to having a role in immunity, is also involved in RANKL-induced osteoclastogenesis. Therefore, it is only logical to assume that the TEC protein may have an important role in relapse (22).

MMPs and their inhibitors tend to increase during tooth movement, peaking a few days later and returning to the control levels within a week (28). There are different types of MMPs, and all of them are responsible for OTM at different phases or for various durations depending on the type and magnitude of force. MMP-9 is responsible for the cleavage of denatured collagen, i.e., gelatin; MMP-13 dissolves native fibrillar collagen; MMP-1 is an interstitial collagenase that hydrolyzes mainly type III collagen;61 and MMP-3 is responsible for the activation of MMPs 8 and 9 (73). The peak for MMP9 and MMP3 ranged from 8 to 72 h after force application. MMP expression is also site specific, although not significant. Bone remodeling is predominantly mediated by the two enzymes ACP and ALP, which are responsible for bone resorption and apposition, respectively. In animal studies, the ACP is increased in the first two weeks, followed by an increase in the ALP in the subsequent couple of weeks, specifically at the tension site. This is followed by a decrease in ALP levels corresponding to hyalinized tissue removal and the initiation of the postlag phase (26). The only long-term human study that evaluated ALP levels postexpansion was by Perinetti et al. in 2015. These patients presented significantly elevated ALP levels at 3 months and 6 months postexpansion. The elevation starts within the first 2–3 weeks, which is also the time taken for bone apposition to begin after a phase of bone resorption. There is no significant difference in the ALP levels at 3 and 6 months (33). Although ALP is one of the most commonly researched biomarkers, it is also frequently associated with inflammation; therefore, an increased ALP level might not be completely attributed to tooth movement. To avoid this, either standardization of oral hygiene regimens should be practiced or split-mouth studies can be designed. TRAP is a potent osteoclast biomarker expressed in areas of compression. TRAP peaked at 4 weeks. LDH peaks around the 14th day and varies depending on the type, magnitude and direction of force. Other inflammatory mediators, such as MPO, which is a sensitive marker for inflammation and pain associated with OTM, showed early increases at 2 h until 1 day (26).

Salivary sample collection is less technique sensitive than GCF collection is. However, across the studies, the time of salivary collection and method of processing differed. The time of salivary sample collection becomes critical because of the spikes in salivary composition attributed to the circadian cycle. During saliva retrieval, care should be exercised to avoid food contaminants that may alter biomarker expression. Most of the studies, however, preferred convenience in collection time. Centrifugation and fast freezing are the most common and accepted methods of processing, and most, if not all, have similar processing methods (29, 74). Proteins can be detected both in saliva and GCF during the active and retentive phases of tooth movement, and authors such as Burke et al. in 2002 have used both sources. One of the benefits of studying biomarkers in GCF is the specificity of the results, but they cannot detect proteins in GCF, which is probably due to the generalized increase in the concentration of protein in GCF (32). Similar results were reported in one of the recent systematic reviews to explore the presence of salivary proteins during OTM by Allen et al. in 2019; interestingly, the most common cytokines were not detectable because they were diluted in saliva or disappeared too quickly before they were detected (29).

Vansant et al. compared and contrasted *in vivo* and *in vitro* studies that assessed the mediators of OTM in their systematic review. Despite the disparity in animal studies, most of them used rodents, which gave some credibility to the results of the studies (27). The results of a 1997 study by King et al. revealed that 13.9 µm of the distal molar moved with time in rats. Interestingly, however, biochemical changes included a sustained increase in bone phosphatases at the mesial roots (previously pressure sites), suggesting continued mesial molar movement after appliance removal. However, this finding does not indicate that there might be continued alveolar bone remodeling several days after removal of force application, which is consistent with the direction of loading of force but returns to baseline levels at the end of 14 days (21).

Vascular endothelial growth factor (VEGF) and endothelin, which mediate vascular alterations and angiogenesis, gradually increase on the tension side during the first week and remain elevated during the second week. Vascularity during relapse was first explained in terms of its number and density by Murell EF et al. in 1996. Changes in vascularity always occur in the direction of tooth movement (either during orthodontic tooth movement or during relapse). They concluded that vascular changes, especially when the orthodontic force is increased after removal, could contribute to relapse by modulating interstitial tissue pressure during alveolar remodeling (31).

There were many confounding factors among the studies, such as patient characteristics (sample size, age and growth status), orthodontic force application (type, time, duration), retention protocol (type, and duration of wear), detection source (GCF or saliva), and oral hygiene maintenance regimen. Although an adequate sample size is more credible to the outcome of results, it is not very critical in orthodontic studies for two reasons: during orthodontic treatment, it makes more sense to monitor a patient for a longer period than to follow up many patients once, and most of the biomarkers have high interpatient variability; therefore, minimizing the sample size and increasing the time intervals to study biomarkers of orthodontic tooth movement will be more logical (29). Depending on growth status, growing and nongrowing individuals express different mediators (25). Heavy orthodontic forces increase ALP levels, indicating that hyalinization is also characterized by increased aspartate transaminase (AST) levels, all of which occur within the first 4 weeks of force application (26).

### Clinical implications

Comprehension of the mediator expression could give an insight to the following;

• Vulnerability of the tissues at various time points and how retention can be tailored accordingly.
• Though presumptive and not yet trial tested, RANKL/OPG, MMP/TIMP modulation may be investigated for reducing the relapse.

### Animal to human studies (translational considerations)

• Saliva is the most common medium used for the assay in humans in comparison with the animal models where pdl/ bone samples are used.
• Oral biotope for humans and animal models cannot be equated
• Experimental forces and remodeling rates differ in animal and human models

## Limitations

• Human studies are exhaustive, and the available literature is skewed toward the study of only some biomarkers, ignoring the remaining biomarkers, increasing the need for more research on several other unexplored mediators of OTM.
• Even with the literature, various results have been reported, which can be attributed primarily to differences in methodologies and participant characteristics, ultimately making comparisons difficult. There was a need to strike the right balance between homogeneity and specificity since any attempt to minimize heterogeneity could lead to population-confined results (29).
• Studies with GCF as a source of biomarkers require excellent handling skills, and only some studies mention the conditions under which the GCF is handled; therefore, the results should be interpreted with caution (26).
• With respect to animal studies, most have used rodents that most closely mimic the biology of humans, thereby making comparisons easier. However, the force levels used are interestingly higher than what is expected. For example, a smaller surface area of the roots of rat molars demands the use of less force to allow for frontal resorptive activity. Therefore, the results from animal studies need to be interpreted with caution (27). Rodents, unlike humans, have a physiological distal drift, and studies involving rodents apply force to the molars mesially, and relapse, if any, is evaluated at the distal aspect of molars. Four animal studies have assessed relapse, and all of them revealed a rapid increase in the rate of distal movement followed by a gradual decline over the next 21 days until it reached rates close to physiological drift, indicating that there was no further relapse. There was also a concurrent increase in the m—RNA expression of *COL1A1,* ALP, and OCN during the initial relapse phase. Franzen et al. demonstrated increased tissue mineral density and % bone volume in the microcts of rats in the direction of relapse at compression sites (20).
• Controlling or standardizing the variables has been easier in *in vitro* studies. However, its clinical translation remains questionable (27).

## Conclusions

### Active phase

#### Inflammatory mediators

There is a release of inflammatory mediators such as cytokines and interleukins within the first hour, reaching a peak approximately 24 h after which they disappear.

#### Osteogenic/enzymatic mediators

ALP and TRAP peak at 1 week and 4 weeks, respectively. OCN and COL1A1 increase later reflecting the consolidation of the newly formed tissues.

#### ECM remodeling mediators

The commonly released enzymes (MMP1 and MMP2) are released within the first 4 h coordinating periodontal ligament and matrix turnover.

### Retentive phase

• The relapse rate is the maximum immediately after the removal of orthodontic force.
• Animal models report an increase in the signalling molecules immediately after unloading, which return to baseline levels within 3 weeks.
• Human cohorts report ALP activity lasting for 3–6 months suggesting an extended period of retention post expansion.
• One of the human studies revealed a distribution of proteins that are detected in both the active and retentive phases, with the exception of tyrosine protein kinase (TEC) which was observed only during the retention phase.

Although there is a consistent temporal pattern of biomarker expression, there is still a lacuna in the mediator specific mechanisms notably during the retention phase. This uncertainty is the key conclusion of our review emphasizing the need for future research focusing both on quantification of relapse and mediator expression during retention phase.

### Knowledge gaps

• Scarce literature on human longitudinal studies during retention phase.
• Limited standardization of matrices, assays and sampling schedules across the existing literature.
• No literature on objective correlation between biomarker expression and the quantitative amount of relapse.

### Scope for future research

• The biomarker studies during retention can adopt standardized time points and correlate the biomarker expression with the amount of relapse measured by the existing relapse indices.
• The evidence on biomarkers can be taken a step further by executing studies that focus on their correlation with the type of archwire, bracket system and microbial colonization.

## Data Availability

The raw data supporting the conclusions of this article will be made available by the authors, without undue reservation.
